# Liberica Coffee (*Coffea liberica*): A Bibliometric Analysis and Targeted Review of Physical, Bioactive, and Sensory Characteristics

**DOI:** 10.3390/molecules31091518

**Published:** 2026-05-02

**Authors:** Muhammad Fakih Kurniawan, A. Ita Juwita, Dian Herawati, Didah Nur Faridah, Nuri Andarwulan, Dominika Średnicka-Tober

**Affiliations:** 1 Division of Food Science and Technology, Faculty of Engineering and Technology, IPB University, IPB Dramaga Campus, Bogor 16680, Indonesia; fakihkurniawan@apps.ipb.ac.id (M.F.K.); a.itajuwita@apps.ipb.ac.id (A.I.J.); dian@apps.ipb.ac.id (D.H.); didah_nf@apps.ipb.ac.id (D.N.F.); 2 South-East Asia Food & Agricultural Science and Technology (SEAFAST) Center, IPB University, IPB Dramaga Campus, Bogor 16680, Indonesia; 3 Department of Food Technology and Nutrition, Faculty of Halal Food Science, Djuanda University, Jl. Tol Ciawi 01, Bogor 16720, Indonesia; 4 Department of Agricultural Technology, Pangkep State Polytechnic of Agriculture, Pangkajene dan Kepulauan 90655, Indonesia; 5 Department of Functional and Organic Food, Institute of Human Nutrition Sciences, Warsaw University of Life Sciences, Nowoursynowska 159C, 02-776 Warsaw, Poland

**Keywords:** Liberica coffee, Excelsa coffee, bibliometric analysis, bioactive compounds, antioxidant activities, sustainable agriculture, agri-food sustainability

## Abstract

Liberica coffee (*Coffea liberica*), including its varieties *C. liberica* var. *liberica* and *C. liberica* var. *dewevrei* (Excelsa), is the third most commercially important coffee species; however, scientific knowledge on its physicochemical, bioactive, and sensory characteristics remains limited compared with Arabica and Robusta. This study evaluates the development of Liberica coffee research and synthesizes current evidence on its key quality attributes. A bibliometric analysis of publications indexed in Scopus, PubMed, and Semantic Scholar was conducted to identify trends, themes, and knowledge gaps, followed by a targeted review of physical properties, bioactive compounds, antioxidant and antibacterial activities, and sensory characteristics. Results show a gradual increase in Liberica research over the past decade, with a shift toward quality attributes and functional properties. Liberica coffee exhibits distinctive physical traits, moderate caffeine levels, and a bioactive profile characterized by chlorogenic acids that vary with processing and roasting, alongside relatively stable alkaloids such as trigonelline and theobromine. The diterpene composition, particularly the kahweol-to-cafestol ratio, distinguishes Liberica varieties. Sensory studies report fruity, jackfruit-like aromas, moderate acidity, and a relatively full body. Despite its potential, standardized data remain limited, highlighting the need for integrated research to support quality differentiation and value addition.

## 1. Introduction

Coffee is one of the most widely consumed beverages in the world and a strategic commodity in the global food industry, contributing significantly to international trade, the economic resilience of farmers, and societal consumption culture [1,2,3]. In recent decades, scientific research related to coffee has rapidly expanded, driven by growing interest in quality differentiation, bioactive compounds, functional potential, sustainability, and consumer sensory perception [3,4,5]. However, most studies have focused on *Coffea arabica* and *Coffea canephora* (Robusta), the two dominant species that collectively account for approximately 95% of global coffee production [6]. As a result, other commercial coffee species still receive relatively less comprehensive systematic scientific attention.

*Coffea liberica* is the third largest commercial coffee species after Arabica and Robusta, primarily cultivated in Southeast Asia and parts of West and Central Africa [7]. This species has distinct morphological characteristics, such as larger fruit and seed sizes, and better resistance to extreme environmental conditions and biotic stress than Arabica [8,9]. Botanically, Liberica coffee is generally classified into two main varieties: *Coffea liberica* var. *liberica* and *Coffea liberica* var. *dewevrei*, widely known as Excelsa [10]. These two varieties exhibit significant differences in plant architecture, seed morphology, chemical composition, and sensory characteristics, resulting in a unique flavor profile distinct from that of mainstream commercial coffees [7,11,12]. In the context of climate change, increasing biotic stress, and the need to diversify premium coffee sources, Liberica coffee is emerging as a strategically important alternative to Arabica and Robusta, yet its scientific characterization remains severely underdeveloped.

Currently, Liberica coffee (*Coffea liberica*), including both var. *liberica* and var. *dewevrei* (Excelsa), is produced in a few tropical countries. Indonesia, the Philippines, and Malaysia are major producers in Southeast Asia. In Indonesia, Liberica coffee is cultivated primarily in Sumatra (Jambi and Riau) and Kalimantan [13,14,15]. In the Philippines, Liberica is known as Barako coffee and holds significant cultural and economic value, while Malaysia, particularly the Johor region, is a major production center for Liberica for the domestic and regional markets [16,17]. Outside Southeast Asia, Liberica is also found in several West and Central African countries, including Liberia, Cameroon, and the Ivory Coast, although its contribution to the global coffee market is still relatively limited compared to Arabica and Robusta [7,18].

Despite its agronomic and sensory uniqueness, the scientific literature on Liberica coffee remains limited and fragmented. Available research generally focuses on specific aspects in isolation, such as genetic diversity, morphological characteristics, or basic physicochemical properties, with a relatively small sample size and a limited geographical scope [8,19,20]. Studies that simultaneously integrate physicochemical characteristics, bioactive compound content, antioxidant activity, and sensory evaluation of Liberica coffee are still rarely reported [11,21,22]. Additionally, the influence of postharvest methods, including natural processing and fermentation based on biological activity, on the quality and functional potential of Liberica coffee has not been systematically reviewed [23,24].

From a bibliometric perspective, the development of Liberica coffee research, including publication productivity, major journals, scientific collaboration patterns, and the evolution of research themes, has not been comprehensively mapped. Bibliometric analysis is a practical quantitative approach for evaluating the dynamics and structure of a research field by analyzing publications and citations [25]. When combined with a targeted critical review, this approach enables the identification of knowledge gaps and the direction of future research in the functional food and beverage industry.

Therefore, this study aims to conduct a bibliometric analysis of scientific publications on Liberica coffee (*Coffea liberica*) indexed in the Scopus database, followed by a targeted review of the physicochemical characteristics, bioactive compounds, antioxidant activity, and sensory properties of Liberica coffee. To the best of the authors’ knowledge, this is the first study to systematically map the research landscape of Liberica coffee using a bibliometric approach while integrating it with a multidisciplinary targeted review. Through data-driven synthesis and critical analysis, this article is expected to serve as a structured scientific reference for researchers and industry practitioners, and to support the enhancement of value addition and diversification of Liberica coffee within the global agri-food system.

## 2. Results and Discussion

### 2.1. Bibliometric Analysis

**Figure 1:**
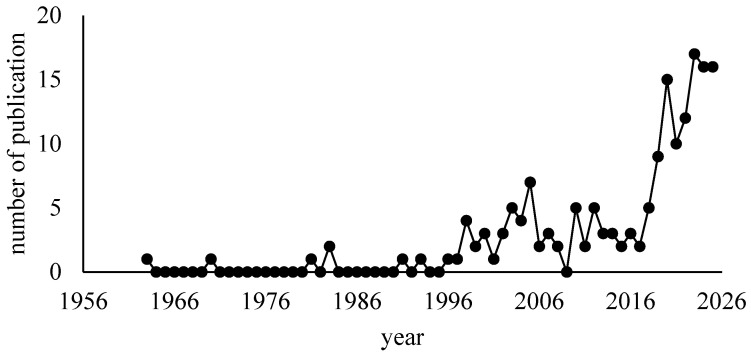
Trends in the number of publications on *C. liberica* by year.

To provide an overview of the temporal development of Liberica coffee research, a bibliometric analysis was conducted based on annual publication output indexed in Scopus. The annual number of publications was analyzed to identify research growth patterns, emerging interest periods, and the evolution of scientific attention toward Liberica coffee over time. The results are presented in Figure 1.

Figure 1 shows that scientific publications on *C. liberica* were minimal until the late 1980s, with almost zero publications per year. An increase was seen from the 1990s through the early 2000s, but it remained fluctuating and relatively low. Significant publication surges occurred only after 2015, with the peak in the 2019–2022 period. This pattern indicates that the *C. liberica* species has long been less in focus of research than Arabica and Robusta, and has received intensive attention only in the last decade. The increasing trend in publications reflects a shift in research interest toward exploring the potential of Liberica, including its chemical characteristics, bioactive compounds, and value-added opportunities, underscoring the urgency of a comprehensive review of the chemical composition of *C. liberica*.

**Figure 2:**
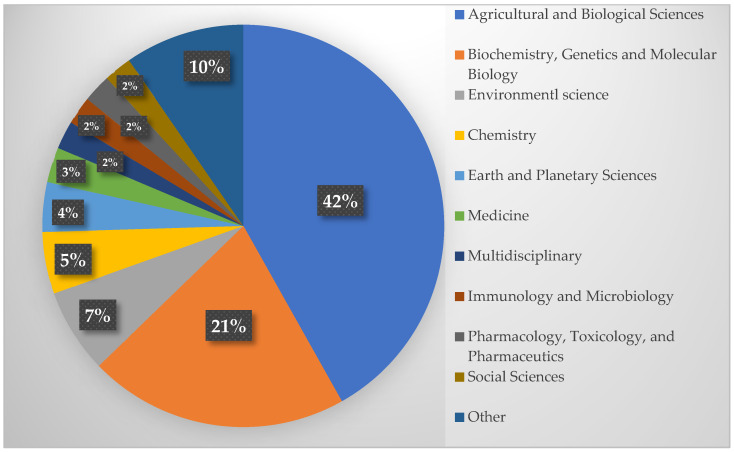
Subject area composition of *C. liberica* research.

Figure 2 shows that publications on *C. liberica* are still dominated by Agricultural and Biological Sciences (42.0%) and Biochemistry, Genetics and Molecular Biology (21.0%), which confirms the strong research focus on cultivation aspects, plant physiology, and the genetic diversity of Coffea [18,19,26,27,28]. The study of microbial ecology and coffee endophytes is also an essential part of Liberica’s basic research [28]. Conversely, the proportions of the Chemistry (5%), Medicine (3%), and Pharmacology, Toxicology, and Pharmaceutics (2%) fields are relatively small, indicating that research on the physicochemical properties, bioactive components, and functional potential of *C. liberica* is still limited, even though these aspects are crucial for the quality, safety, and added value of coffee products. Some studies have begun to reveal the chemical and bioactive properties of *C. liberica*, including its phenolic compound profile, alkaloid content, and responses to postharvest and roasting processes [11,20,29]. However, the number of these studies is still far fewer than that for Arabica and Robusta.

To further understand how this imbalance in research focus has evolved, a keyword co-occurrence overlay visualization was performed. This analysis identifies temporal shifts in dominant research themes, revealing a transition in *C. liberica* studies from genetic–agronomic foundations toward emerging chemical, bioactive, and quality-oriented research topics.

**Figure 3:**
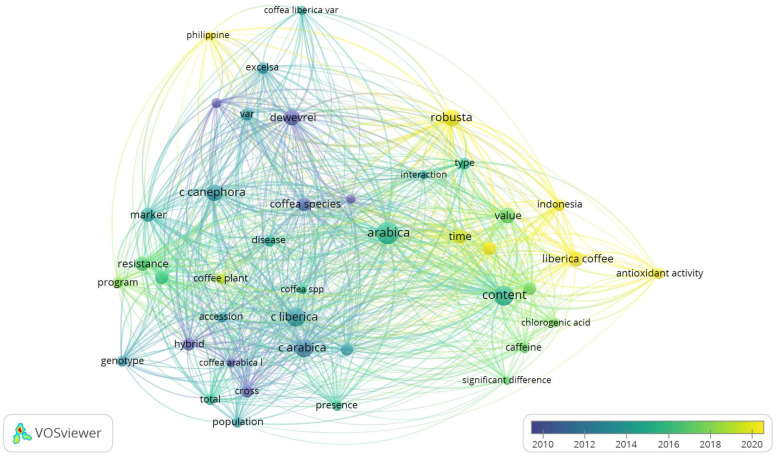
Overlay visualization of keyword co-occurrence in *Coffea liberica* research generated using VOSviewer (version 1.6.20).

This is an overlay network visualization that maps the evolution of *C. liberica* research themes based on keyword co-occurrence and publication year. The color scale indicates a temporal dimension, with blue-purple representing older topics (≈2010–2013), green indicating the transition phase (≈2014–2017), and yellow representing relatively new and developing issues (≈2018–2022) (Figure 3). In the initial phase (blue-purple node), Liberica research was dominated by genetics and breeding clusters, characterized by keywords such as genotype, hybrid, cross, accession, and population, and their relationship with *C. canephora* and *C. arabica*. This pattern indicates that Liberica research in the early period was more oriented toward taxonomic aspects, kinship relationships, and the utilization of Liberica as a source of germplasm for coffee genetic improvement, rather than as a commodity with its own chemical and sensory characteristics [26,27,30].

Entering the transition phase (green node), the research focus shifted toward characterizing the chemical composition and quality of the beans, as evidenced by keywords such as content, chlorogenic acid, caffeine, type, and interaction. This reflects the growing attention being paid to the chemical profile of Liberica, including caffeine and other bioactive components, as determinants of quality and product differentiation [29,31]. In the latest phase (yellow node), the most prominent cluster, strongly connected to *Coffea liberica*, is visible: liberica coffee, antioxidant activity, and time, along with their connection to Indonesia and the Philippines. This pattern indicates a significant shift toward functional studies, particularly antioxidant activity and the bioactive properties of *C. liberica*, thereby confirming Southeast Asia’s role as an emerging hub for modern Liberica research [11,20].

Overall, this map overlay indicates a transformation in the research paradigm for Liberica: from a genetic-taxonomic to a chemical-functional and product-quality approach. However, although the themes of antioxidant activity and caffeine have emerged as current topics, the density of specific chemical nodes (e.g., individual CQAs, diterpenes, and specific Liberica–Excelsa marker metabolites) remains relatively limited compared to Arabica and Robusta [11,29,32]. This pattern reinforces the existence of significant research gaps in physicochemical characterization, detailed bioactive profiles, and their correlation with sensory properties, which serves as the conceptual basis for the targeted review presented in the next section.

Therefore, the following section presents a directed review that specifically summarizes the physicochemical characteristics, bioactive components, and sensory properties of Liberica coffee, including *Liberica* var. *dewevrei* (Excelsa), as a scientific basis for product development and differentiation.

### 2.2. Targeted Review

Liberica coffee cherries exhibited significantly larger dimensions than Arabica and Robusta, particularly in length, width, and thickness, reflecting species-specific morphological traits (Table 1). The larger cherry size of Liberica is associated with its robust plant architecture and thicker pericarp, which may influence moisture retention and drying behavior during postharvest processing [8]. These physical characteristics suggest that Liberica cherries may require tailored postharvest handling strategies to ensure uniform drying and optimal quality development.

Liberica coffee beans possess distinctive physical characteristics that differentiate them from Arabica and Robusta. Bean size, mass, and bulk density are key physical parameters that influence roasting behavior, extraction efficiency, and the release of bioactive compounds. Table 1 presents the main physical dimensions of Liberica coffee beans compared with Arabica and Robusta.

Liberica coffee beans exhibit distinctive physical dimensions compared to Arabica and Robusta, as summarized in Table 1. Liberica beans from Selangor, Malaysia, are substantially larger, thicker, and heavier, with lengths reaching 11.99 ± 1.03 mm, widths 7.67 ± 0.57 mm, and thicknesses 4.67 ± 0.51 mm, exceeding the upper range reported for Arabica beans from Ethiopia and Robusta beans from Kenya [8,22,33,34]. Additional studies also report comparable Robusta bean dimensions from Ghana with lengths, widths, and thicknesses of approximately 9.38 ± 0.63 mm, 6.51 ± 0.50 mm, and 4.28 ± 0.33 mm, respectively, further highlighting variability across origins [35]. The mass of 100 Liberica beans (23.20–25.72 g) is almost double that of Arabica (11–19 g) and substantially higher than Robusta (13.51 g), confirming Liberica as a structurally “giant-bean” coffee type [33,34].

**Table 1:** Physical characteristics of Liberica coffee cherries and beans.

Properties	Liberica Malaysia [8]	Liberica Indonesia [22]	Arabica Ethiopia [34]	Robusta Kenya [33]
Cherries:				
Length (mm)	23.44 ± 1.84	21.72 ± 2.20	15.6 ± 1.05 *	13.40 ± 0.50 *
Width (mm)	20.37 ± 1.62	19.75 ± 2.96	13.70 ± 1.18 *	11.55 ± 0.87 *
Thickness (mm)	18.89 ± 1.40	18.53 ± 2.50	-	-
Volume × 10^−9^ (m^3^)	5500 ± 527.05	5583.86 ± 2116.98	-	-
Beans:				
Length (mm)	11.99 ± 1.03	9.59 ± 0.20	9.38 ± 0.61	9.38 ± 0.71
Width (mm)	7.67 ± 0.57	6.46 ± 0.21	6.08 ± 0.35	6.51 ± 0.56
Thickness (mm)	4.67 ± 0.51	3.54 ± 0.15	4.08 ± 0.29	4.35 ± 0.23
Volume × 10^−9^ (m^3^)	463.37 ± 41.95	465.35 ± 35.78	252.25 ± 41.15	274.14 ± 67.38
Mass of 100 beans	25.72 ± 0.90	23.20 ± 1.27	14.53 ± 2.39	13.51 ± 8.04
Bulk density (kg/m^3^)	677.79 ± 1.49	648.87 ± 26.28	646.88 ± 24.42	744.4 ± 6.78

* [36].

This exceptional morphology implies that Liberica coffee represents a fundamentally different physical system for heat transfer, mass diffusion, and solvent penetration during roasting and brewing processes. The markedly higher bean volume (≈463–465 × 10^−9^ m^3^) compared to Arabica (97–158 × 10^−9^ m^3^) indicates a lower surface-to-volume ratio, which may slow thermal penetration and moisture diffusion during roasting, thereby altering degradation kinetics of thermolabile compounds such as chlorogenic acids and alkaloids [36,37,38].

Although the bulk density of Liberica coffee is comparable to that of Arabica, it is generally lower than that of Robusta, as indicated in Table 1. This suggests that Liberica combines larger physical dimensions with relatively lower compactness compared to Robusta, reflecting inherent structural differences among coffee species. Such characteristics are consistent with previous descriptions of Liberica as a large-seeded coffee type with distinct morphological and structural attributes relative to Arabica and Robusta. These physical distinctions may explain the differences in physicochemical and bioactive behavior observed in Liberica coffee during postharvest processing and subsequent roasting and brewing.

Beyond simple dimensional differences, the physical characteristics of coffee beans play a critical role in determining their behavior during roasting and brewing processes. Previous studies on Arabica and Robusta coffees have demonstrated that bean size, density, and internal structure influence heat transfer rates, moisture migration, and structural expansion during roasting, which in turn affect mass transfer and extraction efficiency during brewing [39,40]. Given the substantially larger bean size, higher volume, and distinct bulk density of Liberica coffee, these physical attributes suggest that Liberica represents a different physical system compared with Arabica and Robusta. Consequently, the physical uniqueness of Liberica beans should be considered a fundamental factor shaping their processing behavior and quality development, rather than merely a morphological distinction, supporting the need for species-specific physical characterization in Liberica coffee research.

Therefore, the physical uniqueness of Liberica beans should be considered a primary determinant of their chemical behavior and functional quality, rather than merely a morphological trait. This reinforces the need for targeted physicochemical and bioactive profiling of Liberica Excelsa coffee as a distinct coffee category, rather than extrapolating Arabica- or Robusta-based processing models.

Due to the scattered and highly heterogeneous nature of Liberica bioactive compound data, a targeted synthesis was conducted to systematically compile quantitative information on chlorogenic acids, alkaloids, and diterpenes across different origins, postharvest processes, and roasting levels. The results are summarized in Table 2.

Although the data are presented in a single table for synthesis, the comparisons in the “Trend” column are based solely on variations within individual studies (intra-study comparisons), rather than on direct comparisons across studies. Given the heterogeneity in extraction methods, analytical techniques, sample types, and reporting units, direct cross-study comparison of absolute values may be misleading. Therefore, the reported values should be interpreted as indicative of general patterns within each experimental context rather than as directly comparable quantitative measures.

**Figure 4:**
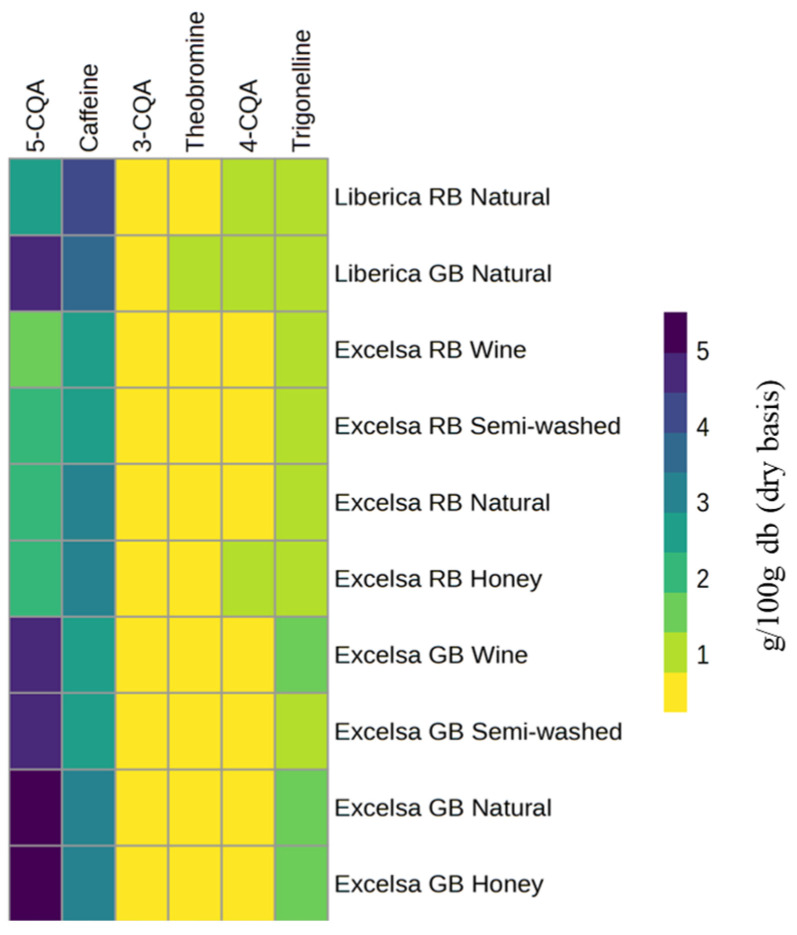
Heatmap visualization of selected bioactive compounds in Liberica and Excelsa coffee based on Table 2. Data for Liberica coffee are from Reference [11], while data for Excelsa coffee are from Reference [20]. RB: roasted bean; GB: green bean.

The chlorogenic acid (CGA) content in *Coffea liberica*, including the Excelsa variety (*C. liberica* var. *dewevrei*), shows substantial variability influenced by genotype, geographical origin, and postharvest processing [11,16]. Table 2 indicates that the total CGA content of green Liberica beans ranges from 0.15 to 6.55 g/100 g. In contrast, roasted coffee ranges from 0.065 to 5.85 g/100 g, depending on roasting degree and brewing or extraction conditions. The 5-CQA isomer dominates the CQA fraction, contributing approximately 60–88% of total CQA. This proportion is comparable to, but often slightly lower than, that reported for Arabica (typically 65–90%) and higher than that of Robusta, indicating that Liberica retains a phenolic profile closer to Arabica while exhibiting its own quantitative signature [44]. Chlorogenic acids, particularly 5-CQA, are widely recognized for their strong antioxidant capacity and have been associated with potential health benefits, including antidiabetic, anti-inflammatory, and cardioprotective effects [45,46]. In addition, a positive correlation between CQA content and antioxidant activity has been reported in Liberica coffee, indicating that higher CQA levels contribute significantly to its overall antioxidant potential [47]. This dominance of 5-CQA and the relative distribution of other bioactive compounds are visually reinforced in Figure 4, where the heatmap highlights consistently higher relative intensity of 5-CQA across both Liberica and Excelsa samples, particularly in green bean forms. In contrast, other CQA isomers and methylxanthines appear at lower, more uniform levels.

Beyond absolute concentrations, the “Trend” column in Table 2 reveals a consistent biochemical response pattern of Liberica bioactive compounds. Across different postharvest pathways, total CQA generally shows a downward trend (↓) after roasting, whereas fermentation treatments tend to produce relatively stable (≈) or slightly increased (↑) CQA levels, depending on the microbial inoculum and moisture regime. This behavior indicates that Liberica CGAs are thermally labile but biologically modulable, a pattern also observed in Arabica but more variable in Liberica, reflecting its broader genetic background and adaptation to marginal tropical environments.

**Figure 5:**
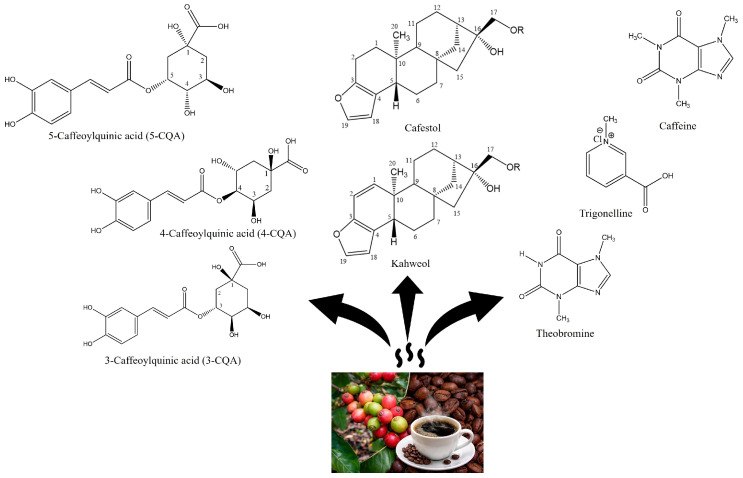
Bioactive compounds in Liberica coffee (The image was adapted from PubChem https://pubchem.ncbi.nlm.nih.gov/ (accessed on 2 March 2026 and redrawn using Chem3D Pro 12.0).

Postharvest processing methods further contribute to the variability of CQA content and composition. Data from *C. liberica* var. *dewevrei* (Excelsa) indicate that different processing techniques, including natural, honey, wine, and semi-washed methods, result in distinct CQA profiles [11]. Among these, honey processing tends to retain or slightly enhance total CQA content, while wine processing is associated with a more pronounced reduction. Semi-washed processing shows intermediate behavior, generally resulting in moderate decreases compared to natural processing. These trends are observed consistently in both green and roasted samples, suggesting that postharvest treatments influence not only the initial CQA composition but also its subsequent transformation during roasting. Such variations may be attributed to differences in microbial activity, fermentation intensity, and moisture conditions, which can affect the stability and extractability of phenolic compounds (Figure 5).

There are eight major CGA isomers in coffee, grouped into caffeoylquinic acids (CQA: 3-, 4-, and 5-CQA), feruloylquinic acids (FQA: 4- and 5-FQA), and dicaffeoylquinic acids (diCQA: 3,4-; 3,5-; and 4,5-diCQA) [48]. Overall abundance typically follows the order 5-CQA > 4-CQA > 3-CQA > 5-FQA > 4-FQA > diCQA. Roasting is the most influential factor altering this CQA profile through thermal degradation and transformation processes [42,49]. The 5-CQA isomer in green beans can be partially converted into 3-CQA and 4-CQA upon heating, thereby reducing the overall CQA concentration in roasted coffee [11]. This roasting-induced decline mirrors that observed in Arabica and Robusta. However, the absolute CGA level of Liberica often remains intermediate between the two species, reinforcing its position as a chemically distinct yet functionally relevant coffee type.

Changes in isomer distribution further illustrate the impact of roasting on CQA composition. Based on studies reporting isomer-specific values in brewed coffee [11,20], 5-CQA, which predominates in green beans, shows the most pronounced decrease following thermal processing. In contrast, the behavior of 3-CQA and 4-CQA is less consistent. In some cases, their relative contribution increases, likely due to isomerization from 5-CQA [11], whereas in others, their concentrations decline alongside 5-CQA, albeit to a lesser extent [20]. These variations suggest that roasting induces both degradation and structural rearrangement of CQAs, while the observed concentrations in brewed samples may also reflect differences in extraction efficiency.

In addition to temperature, fermentation treatment plays a vital role in modifying CQA content. Fermentation using *Bacillus subtilis* in roasted beans produces relatively stable CGA levels (2.96 vs. 2.98 g/100 g of coffee). Still, this process is widely capable of improving flavor scores, optimizing protein and fat content, and minimizing fungal growth [24,50]. Blending strategies with other ingredients, such as *C. zanthorrhiza*, have been shown to effectively modify the flavor and chemical profile. Increasing the percentage of *C. zanthorrhiza* (from 1% to 5%) caused a decrease in total CGA content (from 2.89 to 2.61 g/100 g), indicating that the final CGA composition is primarily determined by the profile of the blending species [51].

Across reported studies using comparable brewing-based analyses, green beans of *Coffea liberica* and its variety *C. liberica* var. *dewevrei* (Excelsa) generally exhibit moderate caffeine contents. Reported values for Liberica and Excelsa green beans typically range from approximately 1.2 to 3.8 g/100 g on a dry basis, depending on origin and processing history [11,20,29]. For comparison, caffeine content in Arabica green beans has been reported to range from 0.78 to 1.55% dry basis in Ethiopian Arabica coffees [52], while Arabica Rinjani from Indonesia contains approximately 1.09 g/100 g [53]. In contrast, Robusta green beans consistently exhibit higher caffeine concentrations, including 2.08 g/100 g in Robusta Lombok [53] and 2.64 g/100 g on a dry basis reported by [54]. Collectively, these data indicate that Liberica and Excelsa occupy an intermediate caffeine position, generally lower than Robusta and overlapping with or slightly higher than Arabica, supporting their potential as coffee types with a moderate and consumer-relevant caffeine profile.

Following postharvest processing, caffeine content in Liberica and Excelsa coffee shows variable but generally declining or stabilizing trends, depending on the processing pathway (Table 2). Fermentation-based treatments, including spontaneous fermentation, controlled microbial fermentation, refermentation with water or coffee cherry extract, and botanical blending, consistently result in caffeine reduction relative to non-fermented counterparts [23,24,41]. In contrast, roasting alone tends to induce either minor increases or relative stabilization of caffeine concentration, likely due to moisture loss and concentration effects rather than de novo caffeine formation [42]. Similar processing-dependent trends were also observed in Excelsa coffee, where wine, honey, and semi-washed processes generally produced lower caffeine levels than natural processing, both in green and roasted beans [11].

In addition to caffeine, trigonelline and theobromine constitute important minor alkaloids in *Coffea liberica* and *C. liberica* var. *dewevrei* (Excelsa). Reported data indicate that trigonelline is present at moderate levels, generally ranging from approximately 0.77 to 1.70 g/100 g on a dry basis in green beans, while theobromine occurs at lower but consistently detectable concentrations, typically between 0.34 and 0.81 g/100 g on a dry basis [11,20]. Together, these alkaloids contribute to the distinctive chemical profile of Liberica-type coffees and are relevant to bitterness perception, sweetness development upon roasting, and the formation of aroma-active compounds, supporting their importance in the overall physicochemical and sensory characterization of Liberica coffee. In addition to their technological and sensory roles, these alkaloids are associated with various physiological effects: caffeine is widely known for its central nervous system stimulation and cognitive-enhancing properties [55,56], trigonelline has been linked to potential antidiabetic and neuroprotective activities [57], while theobromine is associated with mild stimulant and vasodilatory effects [58].

On the other hand, diterpene compounds such as kahweol and cafestol emerge as important chemical markers distinguishing Liberica coffee varieties. Early work by de Roos et al. [32] demonstrated apparent differences in the diterpene profiles of *Coffea liberica* and *C. liberica* var. *dewevrei*, indicating varietal-specific patterns in the composition of kahweol and cafestol. More recent evidence further supports this distinction, showing that *C. liberica* var. *liberica* exhibits a higher kahweol-to-cafestol ratio (0.54–0.56) than var. *dewevrei* (0.16–0.28) [59]. Despite the recognized roles of kahweol and cafestol in cardiovascular health, anti-inflammatory and anticancer activity, lipid metabolism modulation, and their potential application as chemotaxonomic markers, systematic research on diterpene profiles in Liberica coffee remains minimal compared with Arabica and Robusta [60]. This data scarcity highlights a relevant knowledge gap and underscores the need for further investigation into the varietal diversity and functional significance of diterpenes in Liberica coffee.

Collectively, the trend patterns summarized in Table 2 demonstrate that Liberica (*C. liberica* var. *liberica*) and Excelsa (*C. liberica* var. *dewevrei*) possess not only distinct absolute concentrations of bioactive compounds but also characteristic responses to roasting, fermentation, and blending. These response signatures distinguish Liberica from Arabica and Robusta and provide the biochemical rationale for the targeted review presented in the following section, which focuses specifically on the physicochemical, bioactive, and sensory attributes of Liberica and Excelsa coffees. To further contextualize these compositional differences across coffee species, Table 3 presents a comparative summary of major bioactive compounds in Liberica, Excelsa, Arabica, and Robusta coffees.

Table 3 further highlights the comparative distribution of major bioactive compounds across coffee species, as all data are derived from green coffee beans, primarily obtained from naturally processed samples. In addition, the values for chlorogenic acids, trigonelline, theobromine, and caffeine were compiled from coffee samples originating from different regions within Indonesia for Liberica, Excelsa, and Arabica, whereas Robusta data were obtained from literature sources. This provides a comparable context, although variability related to geographical origin, environmental factors, and postharvest practices may still remain. Under these relatively consistent conditions, Liberica and Excelsa coffees exhibit chlorogenic acid levels comparable to or higher than Arabica, while Robusta shows higher but more variable chlorogenic acid content, reflecting the broader compositional range reported in the literature [47].

In terms of alkaloids, Excelsa exhibits higher trigonelline levels than Liberica and Arabica, suggesting a greater potential for the formation of aroma precursors during roasting. Meanwhile, Robusta generally exhibits higher caffeine and theobromine contents, consistent with its stronger bitterness and stimulant intensity, as caffeine is known for its central nervous system stimulation and cognitive-enhancing effects [55]. Differences in diterpene composition further distinguish the coffee species. Arabica shows substantially higher kahweol levels than Liberica, Excelsa, and Robusta, while cafestol concentrations appear more comparable among species.

The bioactivity of coffee results from the interaction of its phytochemical compounds with biological systems that are beneficial to human health. Liberica coffee has been reported to have antioxidant and antibacterial activities, as shown in Table 4. The antioxidant activity of Liberica coffee shows significant variability, determined by interactions among postharvest processes, blending techniques, and thermal parameters during roasting and extraction. However, it should be noted that differences may also influence the reported variability in analytical conditions across studies, including extraction procedures and DPPH assay parameters. The antioxidant capacity of coffee is generally measured using the DPPH (2,2-diphenyl-1-picrylhydrazyl) and FRAP (Ferric Reducing Antioxidant Power) methods, in which lower IC50 values and higher FRAP values indicate greater free radical scavenging activity [22,62]. This pattern confirms that Liberica coffee does not express a single, fixed bioactive profile but rather a highly process-sensitive functional phenotype, in which chemical composition and biological effectiveness are co-determined by postharvest and thermal variables.

Fermentation is one of the postharvest methods that effectively modulates the bioactive profile of Liberica. Data show that refermentation using water or coffee cherry extract media is associated with a reduction in DPPH IC50 values from 40.3 ppm in the natural process to 31.71 ppm and 27.27 ppm, respectively, reflecting an increase in antioxidant activity [23]. Importantly, these comparisons are made within the same study under controlled experimental conditions, thereby minimizing methodological variability. This is in line with the findings of Tarigan et al. [21,24], which indicate that the fermentation process can influence the chemical composition, thereby contributing to the antioxidant capacity of Liberica coffee. In addition to fermentation, a mixing strategy with other functional species, such as *C. zanthorrhiza*, has been reported to result in a pronounced reduction in DPPH IC50 values in a single study. Adding *C. zanthorrhiza* at a 95:5 ratio substantially reduced the DPPH value from 72.12 ppm to 4.98 ppm within the same experimental framework [64]. This sharp increase may suggest a potential synergistic interaction between coffee phenolic compounds and bioactive components from the blending material in neutralizing oxidants. Nevertheless, direct comparison of absolute DPPH IC50 values across different studies should be interpreted with caution due to methodological heterogeneity. This finding positions Liberica not only as a source of intrinsic antioxidants but also as a chemically compatible carrier matrix for external bioactive enrichment, a property that has rarely been reported for Arabica- or Robusta-based formulations.

On the other hand, roasting parameters and brewing methods are determining variables in the final stage of bioactivity. Based on FRAP data, Liberica green coffee beans showed slightly higher antioxidant activity (17.33 TEAC/100 g bk) than roasted beans (17.02 TEAC/100 g bk), suggesting a possible reduction of thermolabile antioxidant compounds during roasting [22]. However, in brewed coffee, dark roasting tends to produce lower DPPH values (72.22 ppm) than light roasting (79.55 ppm), which may be associated with the formation of complex compounds resulting from Maillard reactions, such as melanoidins [42]. These observations are derived from individual studies under specific experimental conditions and should therefore not be directly generalized across different analytical settings. This effectiveness also depends heavily on the extraction method; manual brewing techniques such as V60, French press, and Vietnamese drip produce different DPPH profiles at the same roast level, indicating that the temperature and duration of water-coffee contact significantly affect the yield of bioactive compounds extracted.

Based on the data presented in the table from the study of Latief et al. [64], Liberica coffee from Indonesia shows significant antibacterial activity against *Escherichia coli*. Liberica coffee bean extracts that have been roasted and processed through maceration with ethanol, then fractionated, were tested at concentrations of 10–50%. The test results showed that all fractions could inhibit the growth of *Escherichia coli*. The n-hexane fraction produced an inhibition zone diameter of 2.03–3.63 mm, the ethyl acetate fraction showed the most vigorous activity, with an inhibition zone diameter of 2.9–5.2 mm, and the methanol fraction produced a range of 1.13–5.23 mm. The ethyl acetate fraction consistently showed a higher range of inhibition zones, indicating that this fraction may contain the most effective antibacterial compounds or higher concentrations to combat *Escherichia coli*. These findings underscore that Liberica coffee contains bioactive compounds with antibacterial properties, and that their effectiveness is influenced by the extraction method and solvent used in the fractionation process. However, research on the antibacterial properties of Liberica coffee remains very limited.

Overall, this evidence collectively positions Liberica and Excelsa as emerging functional coffee species whose bioactive potential is not only inherent but also highly engineerable through processing, roasting, blending, and extraction strategies supporting their strategic development beyond the Arabica and Robusta paradigm.

Liberica coffee exhibits a sensory profile that is clearly distinct from Arabica and Robusta, characterized by unique fruity attributes, particularly jackfruit-like aroma, moderate acidity, and a relatively full body (Table 5). Across the reviewed studies, Liberica coffee, especially when roasted light to medium, frequently expresses jackfruit, fruity, and mildly acidic notes, while medium roasting enhances caramel and chocolate attributes, contributing to improved balance and mouthfeel [11,56,58]. Darker roasting levels, on the other hand, tend to increase bitterness and astringency, partially masking the characteristic fruity notes [65]. In contrast, Arabica coffee is commonly associated with floral, citrus-like acidity and delicate sweetness, whereas Robusta typically presents strong bitterness, earthy notes, and a heavier mouthfeel, primarily driven by higher caffeine and phenolic content [63]. These well-established sensory distinctions indicate that Liberica occupies a unique sensory niche that cannot be directly equated with the dominant Arabica Robusta paradigm.

The distinctive sensory characteristics of *Coffea liberica* are closely associated with its volatile composition, which plays a key role in shaping its aroma profile. As summarized in Table 6, the volatile profile of Liberica coffee is dominated by pyrazines, furans, esters, and pyridines, as reported in [20]. These groups of compounds are known to contribute to key aroma characteristics, forming the chemical basis of the sensory profile observed in Liberica coffee.

Table 6 summarizes the main volatile compounds identified in *Coffea liberica* and their associated sensory relevance. The results indicate that pyrazines and furans are the dominant compounds contributing to the characteristic aroma profile. Pyrazines are closely associated with nutty and roasted notes, which correspond to chocolate-related attributes reported in Table 5, while furans contribute to sweet and caramel-like aromas. Pyridine compounds are linked to chocolate-like characteristics, and ester compounds contribute to fruity notes. These fruity-related compounds may collectively explain the distinctive jackfruit-like aroma frequently observed in sensory evaluations [65]. Overall, this relationship demonstrates that the unique sensory profile of Liberica coffee is strongly influenced by its volatile composition.

Fermentation and postharvest processing further modulate the sensory quality of Liberica coffee without fundamentally altering its characteristic profile. Microbial-assisted fermentations have been reported to enhance aroma complexity, flavor clarity, and overall cup score, in some cases upgrading Liberica coffee from premium to specialty classification according to SCAA standards [21,24]. In particular, controlled fermentation processes have been associated with improvements in fragrance, aftertaste, and body, while specific postharvest methods such as honey and wine processing contribute to differences in flavor clarity, balance, and aftertaste complexity [11]. Comparable processing effects have also been observed in Arabica and Robusta coffees, in which fermentation and roasting primarily influence the balance and intensity of sensory attributes rather than altering the inherent species-specific sensory identities [69,70,71,72]. However, compared to Arabica and Robusta, which have been the focus of extensive and standardized sensory research, systematic sensory studies on Liberica and its variety Excelsa remain scarce. Most available reports are localized and limited in scope, highlighting a clear research gap and underscoring the need for broader, standardized sensory evaluations to better position Liberica and Excelsa within the global coffee quality framework.

## 3. Materials and Methods

### 3.1. Bibliometric Analysis

Bibliometric analysis was conducted to map the global research landscape on Liberica coffee (*Coffea liberica*). Bibliographic data were primarily retrieved from the Scopus database due to its broad and consistent coverage of international scientific literature. To enhance the dataset’s comprehensiveness, supplementary searches were conducted using PubMed and Semantic Scholar. The search covered publications from the earliest records available in the selected databases until December 2025. Searches were applied to titles, abstracts, and keywords using the terms: “*Liberica coffee*” *OR* “*Coffea liberica*” *OR* “*Excelsa coffee*” *OR* “*Coffea liberica* var. *dewevrei*”.

Inclusion criteria were defined as studies focusing on Liberica coffee as the main subject, covering physicochemical, bioactive, sensory, postharvest, agronomy, or physiological aspects. Only research articles and review papers published in English or Indonesian were included. Exclusion criteria comprised studies not primarily addressing Liberica coffee, including those focused exclusively on Arabica or Robusta, general studies on genetics, pests, and diseases without specific relevance to Liberica, marginal mentions of Liberica, duplicate records, and publications lacking sufficient primary data. No subject-area restrictions were applied to capture the full interdisciplinary scope of Liberica coffee research. The study selection process, including identification, screening, eligibility, and inclusion stages, is summarized in Figure 2.

The selected records were exported in XLSX and RIS formats. Data in XLSX format were analyzed using the bibliometrix package in RStudio (Version 2024.04.1 Build 748, Posit Software, Boston, MA, USA) to evaluate annual publication trends of *Coffea liberica*. The RIS files were processed using VOSviewer (version 1.6.20, Centre for Science and Technology Studies, Leiden University, The Netherlands) to generate overlay visualizations of keyword co-occurrence, enabling the identification of temporal trends and thematic evolution in Liberica coffee research. The subject area distribution of *Coffea liberica* research was analyzed to identify the disciplinary composition of the retrieved publications. Data were primarily obtained from the Scopus database using the “Analyze Results” feature (document by subject area), which provides standardized classification across multiple research fields. Records retrieved from PubMed and Semantic Scholar, which do not provide a unified subject classification system, were manually categorized based on their research scope and thematic focus, following the same classification framework used in Scopus. The combined dataset was used to generate the subject area composition presented in Figure 2. The visualization was constructed using Microsoft Excel in the form of a pie chart.

### 3.2. Targeted Review Methodology

**Figure 6:**
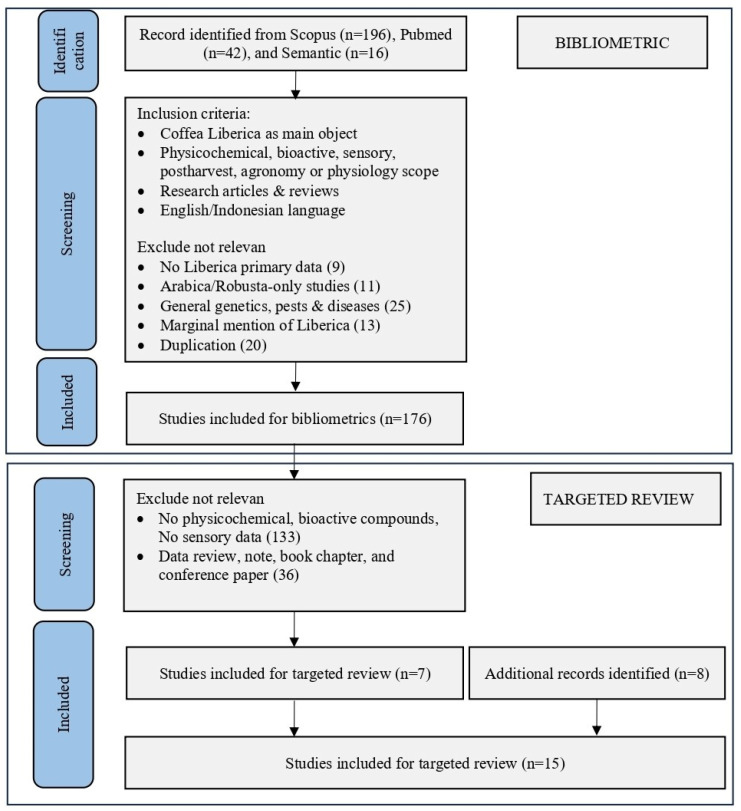
PRISMA flowchart for bibliometrics analysis and targeted review for Liberica coffee.

From the 176 Scopus, PubMed, and Semantic Scholar-indexed articles included in the bibliometric analysis, a second-stage screening was conducted as part of a targeted narrative review to identify studies reporting quantitative data on Liberica coffee (Figure 6). The inclusion criteria for the targeted review were defined as studies reporting physicochemical properties, bioactive compounds, and/or sensory data of *Coffea liberica*, published as peer-reviewed journal articles. To strengthen regionally relevant evidence, nine additional records were identified from Google Scholar and selected Indonesian scientific journals using predefined species-specific keywords (e.g., “Liberica coffee”, “*Coffea liberica*”, and “*Coffea liberica* var. *dewevrei*”). The targeted review focused on physical traits, bioactive compounds, antioxidant and antibacterial activities, and sensory characteristics of Liberica coffee. Studies without extractable quantitative data or without separated Liberica results were excluded. Through this multi-stage screening process, 15 peer-reviewed articles were included in the targeted quantitative synthesis. The list of selected studies is provided in Supplementary Materials Table S1.

Quantitative data extracted from the selected studies were analyzed sequentially according to parameter category to reflect the structure of the results. The analysis first focused on physical characteristics, particularly coffee cherry size and green bean sizes, which are distinctive traits of Liberica and Excelsa coffee. For this specific physical comparison, selected data from *Coffea arabica* and *Coffea canephora* were included solely as comparative references to contextualize the physical attributes of Liberica coffee. They were not incorporated into the primary Liberica dataset.

Following the physical analysis, quantitative data on bioactive compounds were compiled and evaluated. The bioactive components analyzed included chlorogenic acids (CQAs), caffeine, theobromine, trigonelline, and the diterpenes cafestol and kahweol. Reported concentrations were expressed in different units across studies, including g/100 g and mg/g. To ensure comparability, all bioactive compound data were standardized to g/100 g on a sample basis, using the original units. Unit conversion from mg/g to g/100 g was performed using a direct mass-based conversion factor of 0.1. In contrast, values already reported in g/100 g were used without modification. When numerical values were presented only in graphical form, data were extracted using WebPlotDigitizer (https://apps.automeris.io/wpd4/) (accessed on 20 January 2026) and recalculated accordingly. To facilitate comparative visualization of distribution patterns, standardized quantitative data for bioactive compounds were further processed using MetaboAnalyst (https://www.metaboanalyst.ca/home.xhtml) (accessed on 2 February 2026).

Antioxidant activity data were subsequently analyzed according to the analytical method used. Studies using the DPPH assay consistently reported antioxidant capacity as IC_50_ values in ppm, which were compared directly without unit conversion. For studies using the FRAP method, antioxidant capacity values were compiled and compared based on the reported units and experimental conditions, without any mathematical transformation. Antibacterial activity data were synthesized based on inhibition zone diameter measurements. Reported inhibition zone values against different bacterial strains were compiled and compared descriptively, with attention to extraction method, solvent type, and tested microorganism, without further data normalization. Sensory characteristics of Liberica coffee were synthesized narratively rather than quantitatively. Instead of compiling individual sensory attribute scores, a summary table was constructed to highlight consistent sensory trends reported across studies, including dominant aroma descriptors, perceived acidity, body, and overall flavor profile. This approach was adopted to capture the inherent sensory identity of Liberica coffee while avoiding over-interpretation of heterogeneous sensory evaluation protocols.

## 4. Conclusions

This bibliometric analysis and targeted review demonstrate that *Coffea liberica*, including *C. liberica* var. *dewevrei* (Excelsa), represents a distinct yet underexplored coffee species with unique physicochemical, bioactive, and functional characteristics. Bibliometric mapping reveals that Liberica coffee research has gained significant momentum only in the past decade and remains dominated by genetic and agronomic studies, while chemical, bioactive, and functional quality aspects remain comparatively limited. The targeted review highlights that Liberica exhibits distinctive physical bean morphology, intermediate caffeine content relative to Arabica and Robusta, and a characteristic profile of chlorogenic acids, minor alkaloids, and diterpenes. Notably, variability in chlorogenic acids, trigonelline, theobromine, and diterpene composition across varieties and origins suggests strong potential for chemotaxonomic differentiation and modulation of functional quality. Antioxidant and antibacterial activities reported for Liberica further indicate that its bioactivity is not fixed but highly responsive to processing, fermentation, roasting, blending, and extraction strategies. Collectively, these findings position Liberica and Excelsa as emerging functional coffee types rather than marginal alternatives to Arabica and Robusta. However, the synthesis also reveals substantial data gaps, particularly in standardized quantitative profiling, comparative diterpene studies, and the integration of chemical composition with sensory and health-relevant outcomes.

We argue that the current body of literature, while growing, remains insufficient to establish standardized quality benchmarks for Liberica coffee, a gap that this review explicitly identifies as the primary bottleneck to its scientific and commercial advancement. In our view, the most critical limitation of existing studies lies in the lack of harmonized analytical protocols, which makes cross-study comparisons unreliable and hinders the construction of a coherent chemical identity for this species. Furthermore, we recognize that the present review itself is constrained by the heterogeneity of the available data, as the majority of studies originate from a narrow geographic range, predominantly Malaysia and Indonesia, which limits the generalizability of the compositional profiles and bioactivity findings reported here. The absence of integrative studies linking well-defined bioactive concentrations to sensory descriptors and clinically relevant health outcomes represents another critical gap that the authors consider essential to address before Liberica can be credibly positioned as a functional beverage. Future research should prioritize harmonized analytical approaches, broader geographic sampling, and integrative studies linking bioactive compounds to sensory perception and functional performance. Such efforts are essential to support the scientific positioning, value addition, and sustainable development of Liberica coffee within the global coffee and functional beverage landscape.

## Figures and Tables

**Table 2:** Comprehensive compilation of CQAs, alkaloids, and diterpenes in Liberica (*Coffea liberica* var. *liberica* and *C. liberica* var. *dewevrei*) under different processing and roasting conditions.

Chlorogenic Acid (CQA) (g/100 g)															
Variety	Coffee Origin	Type of Bean	Type of Processing	Type of Roasting		Roasting Temperature (°C)	Roasting Time (min)		Type of Sample	3-CQA	4-CQA	5-CQA	CGA	Trend	References
*C. liberica* var. *liberica*	Malaysia	Green bean	-	n.a.		n.a.	n.a.		Brewed	-	-	-	0.16 ± 0.08	≈	[29]
*C. liberica* var. *liberica*	Malaysia	Roasted bean	-	Medium to dark		222–226	14–16		Brewed	-	-	-	0.07 ± 0.05	↓	[29]
*C. liberica* var. *liberica*	Malaysia	Roasted bean	-	Dark		230–234	14–16		Brewed	-	-	-	0.04 ± 0.03	↓↓	[29]
*C. liberica* var. *liberica*	Malaysia	Roasted bean	-	Heavy		235–238	14–16		Brewed	-	-	-	0.02 ± 0.02	↓↓↓	[29]
*C. liberica* var. *liberica*	Indonesia	Roasted bean	Non fermented	Medium		190	12		Methanol Extract	-	-	-	2.98 ± 0.00	≈	[24]
*C. liberica* var. *liberica*	Indonesia	Roasted bean	Fermented *Bacillus subtilis*	Medium		190	12		Methanol Extract	-	-	-	2.96 ± 0.00	↓	[24]
*C. liberica* var. *liberica*	Indonesia	Roasted bean	Not blended	Medium		200 and 230	-		Methanol extract	-	-	-	2.98 ± 0.01	≈	[41]
*C. liberica* var. *liberica*	Indonesia	Roasted bean	Blended *C. zanthorrhiza* (99:1)	Medium		200 and 230	-		Methanol extract	-	-	-	2.89 ± 0.00	↓	[41]
*C. liberica* var. *liberica*	Indonesia	Roasted bean	Blended *C. zanthorrhiza* (97:3)	Medium		200 and 230	-		Methanol extract	-	-	-	2.79 ± 0.00	↓↓	[41]
*C. liberica* var. *liberica*	Indonesia	Roasted bean	Blended *C. zanthorrhiza* (95:5)	Medium		200 and 230	-		Methanol extract	-	-	-	2.61 ± 0.00	↓↓↓	[41]
*C. liberica* var. *liberica*	Indonesia	Green bean	Natural	n.a.		n.a.	n.a.		Brewed	0.75 ± 0.12 *	1.07 ± 0.17 *	4.73 ± 0.55 *	6.55 ± 0.78 *	≈	[20]
*C. liberica* var. *liberica*	Indonesia	Roasted bean	Natural	Medium		195–200	11–12		Brewed	0.53 ± 0.03 *	0.83 ± 0.47 *	2.42 ± 0.13 *	3.78 ± 0.20 *	↓	[20]
*C. liberica* var. *liberica*	India	Roasted bean	Dry	Light		180	9:40		Brewed	-	-	-	5.85 ± 0.00	≈	[42]
*C. liberica* var. *liberica*	India	Roasted bean	Dry	Medium		175	10:25		Brewed	-	-	-	5.08 ± 0.00	↓	[42]
*C. liberica* var. *liberica*	India	Roasted bean	Dry	Dark		202	11:40		Brewed	-	-	-	4.18 ± 0.00	↓↓	[42]
*C. liberica* var. *dewevrei*	Indonesia	Green bean	Natural	n.a.		n.a.	n.a.		Brewed	0.24 ± 0.03	0.45 ± 0.03	5.50 ± 0.14	6.19 ± 0.05	≈	[11]
*C. liberica* var. *dewevrei*	Indonesia	Green bean	Wine	n.a.		n.a.	n.a.		Brewed	0.27 ± 0.05	0.47 ± 0.08	4.47 ± 0.45	5.21 ± 0.50	↓↓	[11]
*C. liberica* var. *dewevrei*	Indonesia	Green bean	Honey	n.a.		n.a.	n.a.		Brewed	0.29 ± 0.04	0.49 ± 0.04	5.44 ± 0.55	6.22 ± 0.60	↑	[11]
*C. liberica* var. *dewevrei*	Indonesia	Green bean	Semi-washed	n.a.		n.a.	n.a.		Brewed	0.29 ± 0.12	0.47 ± 0.12	4.72 ± 0.48	5.48 ± 0.55	↓	[11]
*C. liberica* var. *dewevrei*	Indonesia	Roasted bean	Natural	Light to Medium		200–220	12		Brewed	0.54 ± 0.02	0.75 ± 0.04	2.00 ± 0.07	3.29 ± 0.00	≈	[11]
*C. liberica* var. *dewevrei*	Indonesia	Roasted bean	Wine	Light to Medium		200–220	12		Brewed	0.49 ± 0.08	0.67 ± 0.10	1.75 ± 0.38	2.91 ± 0.00	↓↓	[11]
*C. liberica* var. *dewevrei*	Indonesia	Roasted bean	Honey	Light to Medium		200–220	12		Brewed	0.65 ± 0.04	0.88 ± 0.03	2.29 ± 0.30	3.82 ± 0.42	↑	[11]
*C. liberica* var. *dewevrei*	Indonesia	Roasted bean	Semi-washed	Light to Medium		200–220	12		Brewed	0.56 ± 0.15	0.74 ± 0.15	1.95 ± 0.28	3.25 ± 0.38	↓	[11]
**Alkaloid (g/100 g)**															
**Variety**	**Coffee Origin**	**Type of Bean**	**Type of Processing**	**Type of** **Roasting**		**Roasting Temperature (°C)**	**Roasting Time (min)**		**Type of Sample**	**Trigonelline**	**Theobromine**	**Caffeine**	**Total** **Alkaloids**	**Trend**	**References**
*C. liberica* var. *liberica*	Malaysia	Green bean	-	Medium		n.a.	n.a.		Brewed	-	-	1.23 ± 0.04	-	≈	[29]
*C. liberica* var. *liberica*	Malaysia	Roasted bean	-	Medium		222–226	14–16		Brewed	-	-	1.56 ± 0.03	-	↑	[29]
*C. liberica* var. *liberica a*	Indonesia	Roasted bean	Natural	Medium		200–220	-		Water extract	-	-	1.10 ± 0.11	-	≈	[23]
*C. liberica* var. *liberica*	Indonesia	Roasted bean	Natural, refermented with water	Medium		200–220	-		Water extract	-	-	0.60 ± 0.05	-	↓	[23]
*C. liberica* var. *liberica*	Indonesia	Roasted bean	Natural, refermented with coffee cherry extract	Medium		200–220	-		Water extract	-	-	0.12 ± 0.03	-	↓↓	[23]
*C. liberica* var. *liberica*	Malaysia	Green bean	-	n.a.		n.a.	n.a.		Ethanol extract	-	-	5.40 ± 0.00	-		[43]
*C. liberica* var. *liberica*	Indonesia	Roasted bean	Non fermented	-		190	12		Methanol extract	-	-	0.86 ± 0.01	-	≈	[24]
*C. liberica* var. *liberica*	Indonesia	Roasted bean	Fermented *Bacillus subtilis*	-		190	12		Methanol extract	-	-	0.82 ± 0.10	-	↓	[24]
*C. liberica* var. *liberica*	India	Roasted bean	Dry	Light		180	9:40		Brewed	-	-	1.05 ± 0.00	-	≈	[42]
*C. liberica* var. *liberica*	India	Roasted bean	Dry	Medium		175	10:25		Brewed	-	-	1.12 ± 0.00	-	↑	[42]
*C. liberica* var. *liberica*	India	Roasted bean	Dry	Dark		202	11:40		Brewed	-	-	1.14 ± 0.00	-	↑↑	[42]
*C. liberica* var. *liberica*	Indonesia	Green bean	Natural	n.a.		n.a.	n.a.		Brewed	0.77 ± 0.09 *	0.81 ± 0.12 *	3.77 ± 0.43 *	5.31 ± 0.58 *	≈	[20]
*C. liberica* var. *liberica*	Indonesia	Roasted bean	Natural	Medium		195–200	11–12		Brewed	0.77 ± 0.09 *	0.69 ± 0.05 *	4.23 ± 0.16 *	5.68 ± 0.21 *	↑	[20]
*C. liberica* var. *liberica*	Indonesia	Roasted bean	Not blended	Medium		203	12		Methanol extract	-	-	0.86 ± 0.00	-	≈	[41]
*C. liberica* var. *liberica*	Indonesia	Roasted bean	Blended *C. zanthorrhiza* (99:1)	Medium		203	12		Methanol extract	-	-	0.76 ± 0.04	-	↓	[41]
*C. liberica* var. *liberica*	Indonesia	Roasted bean	Blended *C. zanthorrhiza* (97:3)	Medium		203	12		Methanol extract	-	-	0.66 ± 0.00	-	↓↓	[41]
*C. liberica* var. *liberica*	Indonesia	Roasted bean	Blended *C. zanthorrhiza* (95:5)	Medium		203	12		Methanol extract	-	-	0.56 ± 0.04	-	↓↓↓	[41]
*C. liberica* var. *dewevrei*	Indonesia	Green bean	Natural	n.a.		n.a.	n.a.		Brewed	1.70 ± 0.07	0.34 ± 0.01	3.15 ± 0.02	5.19 ± 0.07	≈	[11]
*C. liberica* var. *dewevrei*	Indonesia	Green bean	Wine	n.a.		n.a.	n.a.		Brewed	1.39 ± 0.35	0.37 ± 0.03	2.56 ± 0.22	4.32 ± 0.5	↓↓	[11]
*C. liberica* var. *dewevrei*	Indonesia	Green bean	Honey	n.a.		n.a.	n.a.		Brewed	1.34 ± 0.05	0.37 ± 0.02	2.88 ± 0.4	4.59 ± 0.5	↓	[11]
*C. liberica* var. *dewevrei*	Indonesia	Green bean	Semi-washed	n.a.		n.a.	n.a.		Brewed	1.28 ± 0.05	0.34 ± 0.02	2.45 ± 0.35	4.07 ± 0.48	↓↓↓	[11]
*C. liberica* var. *dewevrei*	Indonesia	Roasted bean	Natural	Light to Medium		200–220	12		Brewed	1.14 ± 0.03	0.56 ± 0.00	3.13 ± 0.09	4.83 ± 0.00	≈	[11]
*C. liberica* var. *dewevrei*	Indonesia	Roasted bean	Wine	Light to Medium		200–220	12		Brewed	1.17 ± 0.03	0.51 ± 0.03	2.85 ± 0.22	4.53 ± 0.5	↓↓	[11]
*C. liberica* var. *dewevrei*	Indonesia	Roasted bean	Honey	Light to Medium		200–220	12		Brewed	1.28 ± 0.05	0.66 ± 0.02	3.05 ± 0.4	4.99 ± 0.5	↑	[11]
*C. liberica* var. *dewevrei*	Indonesia	Roasted bean	Semi-washed	Light to Medium		200–220	12		Brewed	1.11 ± 0.05	0.6 ± 0.02	2.85 ± 0.35	4.56 ± 0.48	↓	[11]
**Diterpene (g/100 g)**															
**Variety**	**Coffee Origin**		**Type of Bean**		**Type of Processing**			**Method of Analysis**			**Kahweol**		**Cafestol**		**References**
*C. liberica* var. *liberica*	Ivory Coast		Green bean		n.a.			KOH + Diisopropyl ether			0.15 ± 0.00		0.28 ± 0.00		[32]
*C. liberica* var. *dewevrei*	Central African Republic		Green bean		n.a.			KOH + Diisopropyl ether			0.07 ± 0.00		0.48 ± 0.00		[32]

* g/100 g db (dry basis); “-” indicates that data were not reported in the original source; “n.a.” indicates not applicable. The symbol ≈ indicates values comparable to the reference (baseline) within the same study. Arrows indicate relative changes: ↓ slight decrease, ↓↓ moderate decrease, ↓↓↓ substantial decrease, ↑ increase, and ↑↑ substantial increase.

**Table 3:** Comparative concentrations of major bioactive compounds in green coffee beans of Liberica, Excelsa, Arabica, and Robusta coffees (g/100 g dry basis).

Bioactive Compounds	Liberica Jambi [20]	Excelsa Wonosalam [11]	Arabica Enrekang [44]	Robusta [61]
Chlorogenic acids	6.55 ± 0.78	6.19 ± 0.05	5.53 ± 0.12	10.80 ± 6.08
Trigonelline	0.77 ± 0.09	1.70 ± 0.07	1.10 ± 0.02	0.83 ± 0.30
Theobromine	0.81 ± 0.12	0.34 ± 0.01	0.13 ± 0.01	0.30 ± 0.10
Caffeine	3.77 ± 0.43	3.15 ± 0.02	2.80 ± 0.06	4.56 ± 2.21
Kahweol	0.15 ± 0.00 *	0.07 ± 0.00 *	0.69 ± 0.16 **	0.01 ± 0.00 *
Cafestol	0.28 ± 0.00 *	0.48 ± 0.00 *	0.50 ± 0.22 **	0.24 ± 0.00 *

Data for chlorogenic acids, trigonelline, theobromine, and caffeine in Liberica, Excelsa, and Arabica were obtained from coffee samples originating from different regions in Indonesia (Jambi, Wonosalam, and Enrekang), whereas data for Robusta were compiled from published literature sources to represent general compositional ranges. * Diterpene data (kahweol and cafestol) for Liberica, Excelsa, and Robusta were derived from literature sources based on African coffee samples [32]. ** Diterpene data (kahweol and cafestol) for Arabica were compiled from Juwita et al. [59].

**Table 4:** Antioxidant and antibacterial activity of Liberica coffee (*Coffea liberica* var. *liberica* and *C. liberica* var. *dewevrei*) processed using different postharvest treatments.

Antioxidant Activity											
Variety	Coffee Origin	Type of Bean	Type of Processing		Type of Roasting	Type of Sample		DPPH (IC50) (ppm)	FRAP (g TEAC/100 g db)	Trend	References
*C. liberica* var. *liberica*	Indonesia	Roasted bean	Natural		Medium	-		40.3 ± 12.85	-	≈	[23]
*C. liberica* var. *liberica*	Indonesia	Roasted bean	Natural, refermented with water		Medium	-		31.71 ± 16.14	-	↑	[23]
*C. liberica* var. *liberica*	Indonesia	Roasted bean	Natural, referments with coffee cherry extract		Medium	-		27.27 ± 17.92	-	↑↑	[23]
*C. liberica* var. *liberica*	Indonesia	Roasted bean	Unfermented		Medium	Methanol extract		72.12	-	≈	[24]
*C. liberica* var. *liberica*	Indonesia	Roasted bean	Fermentation with *B. subtilis*		Medium	Methanol extract		42.37	-	↑	[21]
*C. liberica* var. *liberica*	Indonesia	Roasted bean	Unfermented		Medium	Methanol extract		34.05	-	≈	[21]
*C. liberica* var. *liberica*	Indonesia	Roasted bean	Fermentation Coffee with *Alcaligenes* sp. 24 h		Medium	Methanol extract		31.74	-	↑↑	[21]
*C. liberica* var. *liberica*	Indonesia	Roasted bean	Fermentation Coffee with *Alcaligenes* sp. 48 h		Medium	Methanol extract		27.37	-	↑↑↑↑	[21]
*C. liberica* var. *liberica*	Indonesia	Roasted bean	Fermentation Coffee with *E. indicum* sp. 24 h		Medium	Methanol extract		33.39	-	↑	[21]
*C. liberica* var. *liberica*	Indonesia	Roasted bean	Fermentation Coffee with *E. indicum* sp. 48 h		Medium	Methanol extract		27.83	-	↑↑↑	[21]
*C. liberica* var. *liberica*	Indonesia	Roasted bean	Not blended		Medium	Methanol extract		72.12 ± 0.00	-	≈	[41]
*C. liberica* var. *liberica*	Indonesia	Roasted bean	Blended *C. zanthorrhiza* (99:1)		Medium	Methanol extract		12.22 ± 0.02	-	↑	[41]
*C. liberica* var. *liberica*	Indonesia	Roasted bean	Blended *C. zanthorrhiza* (97:3)		Medium	Methanol extract		8.39 ± 0.19	-	↑↑	[41]
*C. liberica* var. *liberica*	Indonesia	Roasted bean	Blended *C. zanthorrhiza* (95:5)		Medium	Methanol extract		4.98 ± 0.00	-	↑↑↑	[41]
*C. liberica* var. *liberica*	India	Roasted bean	Dry		Light	Brewed		79.55	-	≈	[42]
*C. liberica* var. *liberica*	India	Roasted bean	Dry		Medium	Brewed		76.44	-	↑	[42]
*C. liberica* var. *liberica*	India	Roasted bean	Dry		Dark	Brewed		72.22	-	↑↑	[42]
*C. liberica* var. *liberica*	Indonesia	Green bean	Natural		n.a.	Brewed		12.91 ± 2.84 *	17.33 ± 1.17	≈	[22]
*C. liberica* var. *liberica*	Indonesia	Roasted bean	Natural		Medium	Brewed		10.57 ± 1.08 *	17.02 ± 1.22	↑	[22]
*C. liberica* var. *liberica*	Indonesia	Brewed	Anaerobic fermented		Light	Vietnam drip		67.34 ± 14.81	-	↑↑	[63]
*C. liberica* var. *liberica*	Indonesia	Brewed	Anaerobic fermented		Light	French press		74.86 ± 8.71	-	↑	[63]
*C. liberica* var. *liberica*	Indonesia	Brewed	Anaerobic fermented		Light	V60		75.7 ± 4.37	-	≈	[63]
*C. liberica* var. *liberica*	Indonesia	Brewed	Anaerobic fermented		Medium	Vietnam drip		87.34 ± 4.77	-	↑	[63]
*C. liberica* var. *liberica*	Indonesia	Brewed	Anaerobic fermented		Medium	French press		97.9 ± 8.97	-	≈	[63]
*C. liberica* var. *liberica*	Indonesia	Brewed	Anaerobic fermented		Medium	V60		73.62 ± 9.27	-	↑↑	[63]
**Antibacterial Activity**											
**Variety**	**Coffee Origin**	**Type of Bean**	**Type of Processing**	**Type of Roasting**	**Type of Sample**		**Type of Bacteria**	**Concentration (%)**	**Inhibition Zone Diameter (mm)**	**Trend**	**References**
*C. liberica* var. *liberica*	Indonesia	Roasted bean	Maceration with ethanol	-	Fraction N-hexane		*Escherichia coli*	10	2.33 ± 0.01	≈	[64]
*C. liberica* var. *liberica*	Indonesia	Roasted bean	Maceration with ethanol	-	Fraction N-hexane		*Escherichia coli*	20	2.03 ± 0.01	↓	[64]
*C. liberica* var. *liberica*	Indonesia	Roasted bean	Maceration with ethanol	-	Fraction N-hexane		*Escherichia coli*	30	2.47 ± 0.01	↑	[64]
*C. liberica* var. *liberica*	Indonesia	Roasted bean	Maceration with ethanol	-	Fraction N-hexane		*Escherichia coli*	40	3.63 ± 0.01	↑↑↑	[64]
*C. liberica* var. *liberica*	Indonesia	Roasted bean	Maceration with ethanol	-	Fraction N-hexane		*Escherichia coli*	50	3.17 ± 0.01	↑↑	[64]
*C. liberica* var. *liberica*	Indonesia	Roasted bean	Maceration with ethanol	-	Fraction Ethyl Acetate		*Escherichia coli*	10	5.20 ± 0.01	≈	[64]
*C. liberica* var. *liberica*	Indonesia	Roasted bean	Maceration with ethanol	-	Fraction Ethyl Acetate		*Escherichia coli*	20	2.90 ± 0.01	↓↓↓↓	[64]
*C. liberica* var. *liberica*	Indonesia	Roasted bean	Maceration with ethanol	-	Fraction Ethyl Acetate		*Escherichia coli*	30	3.3 ± 0.01	↓↓↓	[64]
*C. liberica* var. *liberica*	Indonesia	Roasted bean	Maceration with ethanol	-	Fraction Ethyl Acetate		*Escherichia coli*	40	3.70 ± 0.02	↓↓	[64]
*C. liberica* var. *liberica*	Indonesia	Roasted bean	Maceration with ethanol	-	Fraction Ethyl Acetate		*Escherichia coli*	50	4.27 ± 0.02	↓	[64]
*C. liberica* var. *liberica*	Indonesia	Roasted bean	Maceration with ethanol	-	Fraction Methanol		*Escherichia coli*	10	1.13 ± 0.01	≈	[64]
*C. liberica* var. *liberica*	Indonesia	Roasted bean	Maceration with ethanol	-	Fraction Methanol		*Escherichia coli*	20	2.30 ± 0.01	↑	[64]
*C. liberica* var. *liberica*	Indonesia	Roasted bean	Maceration with ethanol	-	Fraction Methanol		*Escherichia coli*	30	3.33 ± 0.01	↑↑	[64]
*C. liberica* var. *liberica*	Indonesia	Roasted bean	Maceration with ethanol	-	Fraction Methanol		*Escherichia coli*	40	4.50 ± 0.01	↑↑↑	[64]
*C. liberica* var. *liberica*	Indonesia	Roasted bean	Maceration with ethanol	-	Fraction Methanol		*Escherichia coli*	50	5.23 ± 0.01	↑↑↑↑	[64]

* mg/mL; “-” indicates that data were not reported in the original source; “n.a.” indicates not applicable. The symbol ≈ indicates values comparable to the reference (baseline) within the same study. Arrows indicate relative changes: ↓ slight decrease, ↓↓ moderate decrease, ↓↓↓ substantial decrease, ↓↓↓↓ drastic decrease. ↑ slight increase, ↑↑ moderate increase, ↑↑↑ substantial increase, ↑↑↑↑ drastic increase.

**Table 5:** Summary of sensory characteristics of *Coffea liberica* reported in the literature.

Variety/ Country	Processing/Treatment	Sensory Methods	Key Sensory Attributes	References
*C. liberica* /Indonesia	Anaerobic fermentation, various roasting & brewing	Quantitative descriptive analysis	Jackfruit-like aroma (light roast), fruity and acidic notes; chocolate and caramel at medium roast; fuller body	[63]
*C. liberica* /Indonesia	Fermentation with *Alcaligenes* sp. and *E. indicum* (24–48 h), light–dark roasting	Cupping test	Generally high cupping scores (≈7.5–8.0 per attribute); fermentation improved fragrance, flavor, aftertaste, body, and overall score; medium-roast fermented samples showed the best overall cup quality	[21]
*C. liberica* /Indonesia	Wet fermentation using *Bacillus subtilis* (fermented vs. unfermented Liberica coffee)	Cupping test	Fermented Liberica coffee (FLC) showed higher scores across almost all sensory attributes than original Liberica coffee (OLC); final score ≈86 classified as specialty coffee, while OLC (<80) was categorized as premium	[24]
*C. liberica* /Indonesia	Honey process; light–dark roasting; V60 & French press	Quantitative descriptive analysis & hedonic test	Jackfruit, fruity, caramel, chocolate, smoky; light roast–V60 most preferred; dark roast increases bitterness & astringency	[65]
*C. liberica* var. *dewevrei* /Indonesia	Natural, semi-washed, wine processing	Cupping test	Nutty, caramel, chocolate flavors; balanced acidity; clean and complex aftertaste (wine process)	[11]

**Table 6:** Main volatile compounds and their sensory relevance in *Coffee liberica* var. *liberica*.

Compound	Chemical Class	Sensory Descriptor	Related Sensory Attribute (Table 5)	Reference
2-Methylpyrazine	Pyrazine	Nutty, roasted	Nutty, chocolate	[66,67]
2,6-Dimethylpyrazine	Pyrazine	Roasted, cocoa-like	Chocolate	[11,66]
3-Ethyl-2,5-dimethylpyrazine	Pyrazine	Nutty, roasted	Nutty	[67]
Furfural	Furan	Sweet, caramel-like	Caramel	[66,67]
5-Methylfurfural	Furan	Caramel, sweet	Caramel	[11,66]
2-Furanmethanol	Furan	Sweet, caramel-like	Caramel	[67]
Pyridine	Pyridine	Bitter, chocolate-like	Chocolate	[66]
Ethyl salicylate	Ester	Sweet, fruity	Fruity	[68]
Methyl salicylate	Ester	Minty, wintergreen	Fruity/complex	[68]
Isovaleric acid	Acid	Pungent, strong aroma	Fermented/strong notes	[11,67]

## Data Availability

The contributions presented in this study are included in the article. Further inquiries can be directed to the author, Nuri Andarwulan.

## Supplementary Materials

The following supporting information can be downloaded at: https://www.mdpi.com/article/10.3390/molecules31091518/s1, Table S1: Studies included in the targeted literature review and quantitative synthesis of Liberica coffee.
