# Supplementary material for: Liberica Coffee (Coffea liberica): A Bibliometric Analysis and Targeted Review of Physical, Bioactive, and Sensory Characteristics
Source: Molecules. 2026 May 2;31(9):1518. doi: 10.3390/molecules31091518 (PMC13164717; doi:10.3390/molecules31091518)
Supplement: Supplementary file 1 [file molecules-31-01518-s001.zip › molecules-4229736-supplementary.pdf]

Table S1. Studies included in the targeted literature review and quantitative synthesis of Liberica coffee.

No.	References
1.	D Herawati, MO Loisanjaya, RH Kamal, DR Adawiyah, and N Andarwulan. Profile of bioactive compounds, aromas, and cup quality of Excelsa coffee (*Coffea liberica* var. *dewevrei*) prepared from diverse postharvest processes. *International Journal of Food Science* 2022. https://doi.org/10.1155/2022/2365603.
2.	D Hanifah, D Herawati, and N Andarwulan. Effects of roasting on profiles of non-volatile and volatile compounds in Liberica coffee from Jambi, Indonesia. *International Food Research Journal* 2025. https://doi.org/10.47836/ifrj.32.1.12.
3.	A Mubarak, KD Croft, CB Bondonno, and NS Din. Comparison of Liberica and Arabica coffee: chlorogenic acid, caffeine, total phenolic and DPPH radical scavenging activity. *Asian Journal of Agriculture and Biology* 2019; 7(1):130–136.
4.	B de Roos, G van der Weg, R Urgert, and P van de Bovenkamp, A Charrier and MB Katan. Levels of cafestol, kahweol, and related diterpenoids in wild species of the coffee plant *Coffea*. *Journal of Agricultural and Food Chemistry* 1997. https://doi.org/10.1021/jf9700900.
5.	S Septiana, T Mahatmanto, A Salsabila, and WB Sunarharum. The effect of roasting degrees and brewing techniques on the physicochemical and sensory characteristics of anaerobically fermented Liberica coffee (*Coffea liberica*). *Trends in Sciences* 2025. https://doi.org/10.48048/tis.2025.9819.
6.	M Latief, H Heriyanti, IL Tarigan, and S Sutrisno. Preliminary data on the antibacterial activity of *Coffea arabica*, *Coffea canephora* and *Coffea liberica*. *Pharmacognosy Journal* 2022. https://doi.org/10.5530/pj.2022.14.53.
7.	IL Tarigan, Z Adriliana, A Adiningtyas Putri, N Ariefandie Febrianto, M Latief, and S Sutrisno. Changes in the chemical compound and sensory profiles of Liberica fermentation (*Coffea liberica*) with cellulolytic bacteria *Alcaligenes* sp. and *Exiguobacterium indicum*. *Coffee Science* 2024. https://doi.org/10.25186/.v19i.2258.
8.	I Ismail, IA Anuar, M Shamsul, and Rosnah. Physical properties of Liberica coffee (Coffea liberica) berries and beans. *Pertanika Journal of Science & Technology* 2014; 22(1), 65–79.
9.	D Hanifah, N Andarwulan, and D Herawati. Karakteristik fisikokimia dan kapasitas antioksidan kopi Liberika dari Kabupaten Tanjung Jabung Barat, Jambi. *Jurnal Teknologi dan Industri Pangan* 2022. https://doi.org/10.6066/jtip.2022.33.1.39.
10	WB Sunarharum, HR Umami, AA Kartika, S Septiana, and T Mahatmanto. Re-fermentation of green Liberica coffee (Coffea liberica) beans: impact on the caffeine and antioxidant content of the roasted beans. *Journal of Experimental Life Sciences* 2023.https://doi.org/10.21776/ub.jels.2023.013.02.001.
11.	IL Tarigan, E Aulia, H Heriyanti, M Latief, and S Sutrisno. Enhancement of Liberica coffee quality by wet fermentation using *Bacillus subtilis*. *Alchemy* 2024. https://doi.org/10.20961/alchemy.20.2.74248.162-177.
12.	T Vanathi, HR Bhoomika, CS Ravi, RM Patil, and MS Nagaraja. Effect of coffee species and roasting levels on biochemical composition and antioxidant properties. *International Journal of Advanced Biochemistry Research* 2025. https://doi.org/10.33545/26174693.2025.v9.i10Si.5914.
13.	BV Li Qi and E Nillian. Caffeine extraction from Sarawak Liberica coffee. *Journal of Coffee and Sustainability* 2024. https://doi.org/10.21776/ub.jcs.2024.01.01.04.
14.	M Latief, RW Muntasir, DE Wijaya, IL Tarigan, and S Sutrisno. Synergetic effects of *Coffea liberica* and *Curcuma zanthorrhiza*: study of sensory profile, proximate, and chemical compound. *Beverages* 2025. https://doi.org/10.3390/beverages11010009.
15.	WB Sunarharum, M Nurminah, and NG Purba. Effect of different roasting levels and manual brewing techniques on the sensory profile of Liberica coffee with honey process. *Journal of Coffee and Sustainability* 2024. https://doi.org/10.21776/ub.jcs.2024.01.01.05.
